# “The intervention piece…that's still the hardest part of it all.” Enhancing brief intervention skills among public health nurses

**DOI:** 10.1186/1940-0640-10-S2-P9

**Published:** 2015-09-24

**Authors:** Rebecca Porter, B Hanson, S Faulkner, D King

**Affiliations:** 1 Center for Behavioral Health Research & Services, University of Alaska Anchorage, Anchorage, USA

## Background

Nurses are likely to encounter opportunities to engage patients in alcohol interventions, yet few have received preparation for this work. Although screening procedures are relatively straightforward and easily utilized by nurse professionals, developing a personalized, brief, and effective response to promote behavior change for indicated cases is a clinical skill that, for many nurses, requires deliberate effort to acquire[1,2]. To support development of alcohol screening and brief intervention (SBI) skills among nurses and implement alcohol SBI as a routine practice, Alaska Public Health Nursing (PHN) collaborated with the Arctic FASD Regional Training Center at the University of Alaska Anchorage.

The objective is to describe ongoing training needs for nurses conducting alcohol SBI.

## Material and methods

The university team and PHN leadership jointly planned policies and procedures for alcohol SBI implementation and PHN staff were trained. PHN shared quantitative tracking data and the university team collected qualitative data from contact notes and interviews to assess implementation processes and identify ongoing needs. Findings were iteratively discussed by the university team, presented to PHN leadership, and used to refine processes.

## Results

Initial findings revealed nurse confidence and ability to conduct alcohol screening along with nurse hesitation and challenges with conducting brief interventions. The university team identified a need for additional nurse education and provided follow-up training and clinical tools specific to brief intervention skills. Following additional training and practice, nurses reported increased understanding of brief intervention components and importance. Nurses identified clinical tools as helpful resources to support adoption and, over time, nurses demonstrated increased confidence and ability to conduct both screening and brief intervention activities.

## Conclusions

With planning, training, and commitment to continuous improvement, public health nurses were able to consistently incorporate alcohol SBI as a new practice. Follow-up training and customized resources proved to be important components for brief intervention skill development among nurses.
